# Opinions and experiences on the provision of care to people with mental illnesses: a qualitative study with Doctor of Pharmacy graduates after a rotation in psychiatry

**DOI:** 10.1007/s11096-023-01646-1

**Published:** 2023-10-05

**Authors:** Monica Zolezzi, Rawan Ghanem, Shahd Elamin, Yassin Eltorki

**Affiliations:** 1 College of Pharmacy, QU Health, Qatar University, Doha, Qatar; 2 Mental Health Services, Hamad Medical Corporation, Doha, Qatar

**Keywords:** Doctor of pharmacy, Mental health, MESH [mental disorders], PharmD, Psychiatric rotations, Stigma

## Abstract

**Background:**

Pharmacists are vital to optimizing therapy of people with mental illnesses. Limited knowledge, lack of confidence, and mental health-related stigma can lead to pharmacists’ reluctance in the provision of pharmaceutical care to this population. Advanced pharmacy practice experiences (APPE) in mental health have been reported as valuable learning opportunities to overcome these challenges.

**Aim:**

This study aimed to explore PharmD graduates’ perceived preparedness, attitudes, beliefs, and opinions on influencing factors for the provision of pharmaceutical care to people with mental illnesses after completing an APPE rotation in psychiatry.

**Method:**

All PharmD graduates who had completed a rotation in psychiatry were invited to participate in semi-structured interviews. An interview guide was developed based on a literature review. A total of 11 PharmD graduates agreed to participate in the interviews, which were recorded, transcribed, and analysed inductively using thematic analysis and following a phenomenological approach.

**Results:**

Five themes were identified: Prior familiarity to mental health, opinions on the rotation, views on stigma, rotation’s areas of improvement, and the positive impact of the rotation on practice. Although participants started the psychiatric rotation with low confidence and a sense of apprehension, they described their experience as unique, eye-opening, and insightful. Familiarity with mental health conditions before the rotation were perceived as a challenge to achieving full confidence in mental health care provision.

**Conclusion:**

For the most part, the APPE in psychiatry was viewed as a positive opportunity for enhancing the PharmD graduates’ insight, knowledge, and skills for pharmaceutical care provision to people with mental illnesses.

**Supplementary Information:**

The online version contains supplementary material available at 10.1007/s11096-023-01646-1.

## Impact statements

- This study shows that clinical rotations in psychiatry play an important role in breaking down stigma and misconceptions, as well as increasing confidence of PharmD graduates when providing care to people with mental illnesses.
- Despite the growing awareness about mental health, PharmD students reported starting their psychiatric rotations with fear and apprehension.
- Longer training in managing the diversity of psychiatric conditions was identified as a necessity to achieve full confidence when providing pharmaceutical care to people with mental illnesses.

## Introduction

Pharmacists have a vital role in managing people with mental illnesses. They are responsible for optimizing patients’ drug therapy, educating them on their medications, and ensuring their safe and effective use. Multiple systematic reviews have demonstrated a positive impact of pharmacists’ roles in mental health across multiple countries and settings (community, primary care, and specialized psychiatric facilities), including improvement in medication adherence, prescribing practices, and satisfaction among people with mental illnesses [1–3].

This valuable role can be affected negatively by many factors, such as mental health stigma, pharmacists’ limited knowledge and experience managing people with mental as opposed to those with physical illnesses. In a study conducted in the United States of America (USA), which assessed community pharmacists’ attitudes towards service provision to patients with severe/persistent mental illnesses, 72% of the participants stated they lacked confidence in providing pharmaceutical care to this population (e.g., counselling and monitoring for safety/efficacy of psychiatric medications) [4]. In another study in the USA, a positive correlation was found between community pharmacists’ lack of previous experience managing patients with mental health disorders with lack of confidence and reduced levels of comfort when providing care [5]. Similarly, in a Malaysian study, about 50% of the cohort of community pharmacists reported inadequate knowledge about mental illnesses as the biggest barrier to care provision in mental health [6]. Considering the present study was conducted in Qatar, it is also important to highlight that within the Arab culture, stigmatizing attitudes towards people with mental illnesses have been extensively reported in the literature [7], and may further impact the pharmaceutical care provision to this population in this area of the world.

For the pharmacy profession to overcome these issues hindering pharmaceutical care provision to people with mental illnesses, many pharmacy schools have incorporated mental health education into the curricula to reduce mental health-related stigma among students [8, 9]. Advanced Practice Pharmacy Experiences (APPEs), commonly referred to as "rotations", allow student pharmacists to gain experience, apply and build on previous knowledge and skills drawn from their didactic learning, and gain professional competence and confidence in delivering pharmaceutical care by completing clinical experiences under the supervision of preceptors [10]. The PharmD programme at the College of Pharmacy, Qatar University (CPH-QU) was initiated in 2011. It is a post-baccalaureate programme that integrates APPEs into its experiential education curricula where the students attend a diversity of clinical rotations at different sites. The psychiatric PharmD rotation was first offered in 2015, to improve the confidence and skills of PharmD graduates in pharmaceutical care provision to people with mental illnesses.

Previous efforts have been conducted to study experiences in pharmacy mental health rotations. For instance, in the USA, Cates and Woolley surveyed pharmacy students’ attitudes towards suicide prevention and care provision to patients with depression and schizophrenia, before and after their mental health rotation [11]. While the authors deemed the rotation to have caused no major change on students’ social distance from patients with mental illness, they reported an overall improvement in the students’ attitudes towards schizophrenia, suicide prevention, and pharmaceutical care provision in this population [11]. More recently, similar studies conducted in the USA and Nigeria showed mixed findings on the benefits of mental health rotations on the students’ attitudes towards people with mental illnesses [12, 13]. However, there is limited qualitative research on PharmD graduates’ opinions and perceived preparedness for the provision of pharmaceutical care to this population after completing a clinical rotation in psychiatry.

### Aim

This study aimed to explore PharmD graduates’ experiences in an APPE rotation in psychiatry and its impact on their attitudes, beliefs, and opinions on influencing factors for the provision of pharmaceutical care to people with mental illnesses. It also aimed to investigate the graduates’ preparedness for the psychiatric rotation, and the educational gaps that needed to be addressed.

### Ethics approval

This study was approved by the QU Institutional Review Board (IRB) on January 20, 2021, approval number: QU-IRB-1465-EA/21.

## Method

### Study design

This project used a qualitative phenomenological approach to explore CPH-QU PharmD graduates’ experiences during their psychiatric APPE rotation through semi-structured interviews. This methodology was used to allow for rich discussions and deep understanding of the graduates’ perceptions and attitudes and their effect on their present and future care provision to people with mental illnesses [14].

### Study participants

All 20 CPH-QU PharmD graduates who had completed an APPE in psychiatry during their PharmD programme between the academic years of 2015 to 2020, were invited to participate. Graduates were targeted for this study, as opposed to students, to capture the impact of the psychiatric rotation on the participants’ practice post-graduation. This allowed for potentially more participants since only a few students complete a rotation in psychiatry each year (1–3 students). Two sets of email invites including informed-consent sheets were sent to the graduates’ alumni emails. Sampling continued until data saturation was reached, defined as the point at which emerging concepts from three consecutive unique interviews revealed no additional themes [15, 16].

### Guide development and data collection

To guide the interviews, questions were designed by undergraduate student researchers based on an extensive literature review (Supplementary material), and then piloted by the principal investigator (PI) to obtain information about the following topics:

i. PharmD graduates’ familiarity with mental illnesses prior to the mental health rotation.
ii. Graduate’s preparedness before the rotation.
iii. Differences and similarities of the rotation when compared to other rotations offered in the programme.
iv. Mental health-related stigma.
v. Influence of the APPE rotation in psychiatry on participants’ current practice.
vi. Challenges during the specialized APPE rotation.
vii. Needs for improving the rotation.

Following the COVID-19 pandemic’s health precautions, thirty-minute interviews were carried out online on Microsoft Teams®. Two undergraduate researchers (RG, MS) conducted the interviews February-April 2021, until theme saturation was achieved. The student researchers received training in qualitative research through an earlier undergraduate course in research and piloted the guide with the (PI) (MZ). They had no established relationships with the participants. Interviews were recorded through Microsoft Teams® with access limited to the team. They were then manually transcribed verbatim and transferred to an Excel datasheet for analysis. Informed consent was collected from participants beforehand by email and reconfirmed at the interview.

### Data analysis

Interview analysis was guided by Sutton and Austin’s qualitative analysis principles, which provide insight into data interpretation and analysis of interviews with patients or pharmacists [17]. As recommended for conducting qualitative research [18, 19], interview transcripts were anonymized, collaboratively coded by RG and MS, then checked with a third investigator (MZ). Codes were classified, inserted into the Excel datasheet, then used to inductively extract themes. Consensus was reached through discussion and agreement between the two coders and three investigators (MZ, SE and YE).

### Trustworthiness

To enhance this study’s rigor, Guba and Lincoln’s four criteria for qualitative studies were applied [20]. Credibility was ensured through purposive sampling, adequate interview time, and peer debriefing with a third investigator. To support data consistency over time and establishing clear derivation of results from the data collected, dependability and confirmability were ensured by clear documentation of the study’s methods, quote provision as links to raw data, and an extra review of the themes identified by a fourth investigator (SE) [20, 21].

## Results

Eleven semi-structured interviews were conducted with CPH PharmD graduates who completed an APPE rotation in psychiatry. As summarized in Table 1, participants were mainly female (90.9%) and for the most part graduated in 2017. This reflects the programme graduates’ demographics as most students enrol directly from an all-female Bachelor-of-Science Pharmacy programme at QU. Table 1 illustrates the sample’s demographics.

**Table 1:** Participants' demographics

	N (%), P#
*Gender*	
Male	1 (9.1%)
Female	10 (90.1%)
*Graduation year*	
2016	1 (9.1%)—P10
2017	4 (36.4%)—P01, P02, P06, P08
2018	2 (18.2)—P04, P05
2019	2 (18.2%)—P03, P11
2020	2 (18.2%)—P07, P09

N = Total number of participants; % = percent; P# = Participant code

Five major themes emerged from the transcript analysis: Familiarity with mental illnesses prior to their mental health rotation, opinions on the rotation, mental health-related stigma, barriers hindering graduates’ care provision to patients with mental, and the impact of the rotation on their practice.

### Theme 1: Familiarity with mental illnesses before the rotation

#### Undergraduate experiential learning

Some PharmD graduates revealed gaining experience in mental health practice during their undergraduate experiential training, also referred to as Structured Practical Experiences in Pharmacy (SPEP). Participants mentioned that encounters with patients during SPEP rotations at the psychiatric hospital or other hospitals offered them the opportunity to become familiar with some basic mental-health concepts.

*“My PharmD wasn't my first mental health rotation. I also went before in my undergrade training so in PharmD. I had an idea.” P10*

#### Personal experiences

Some graduates reported personal experiences with people with mental illnesses, which deepened their understanding of these disorders.

*“It was a bipolar patient, one friend of the family. Another encounter… we saw a teenager… we were really really afraid and anxious… after that I really understood what was going on with that patient and what he did and how really his family are suffering.” P09*

#### Undergraduate curriculum

For the most part, PharmD graduates perceived their undergraduate curriculum’s mental health component provided them with knowledge of some psychiatric illnesses, psychopharmacology, and pharmacotherapy, but felt it was insufficient engaging them with persons with lived experience of mental illness or managing the diversity of mental health related presentations. Participants thought that during the rotation, they often needed to review basic concepts in mental health practice, and felt unfamiliar with many psychiatric presentations. They reported that the undergraduate curriculum does not provide them with opportunities to meet real patients, and thus felt unprepared to deal with emotionally challenging situations.

*“I felt like the college (tried) to train us (on) how to deal with psychiatric patients emotionally. But of course… seeing it for the first time in real-case scenarios… and seeing how much mental illness affects patients and their lives and their families’ lives was a different thing.” P06*

### Theme 2: Graduates’ opinions of the mental health rotation

#### Experiences early in the rotation

For the most part, participants mentioned they were keen to learn about mental illnesses and have the opportunity to meet and care for patients receiving mental health services.

*“OK, so actually I was initially interested, like even before. I'm still interested in mental health.” P07*

Some participants also voiced feeling nervous and even afraid of the patients at the beginning of their psychiatric rotations, although this eased off as the rotation progressed. Some graduates were emotionally affected, feeling helpless when managing patients. This caused some students to feel unprepared or not confident when counselling patients or managing drug therapy, especially at the beginning of the rotation.

*“I was a little bit scared of patients... some of them already admitted to crimes… Some of them are quite schizophrenic patients…” P03*

*“It was so depressing for me to see such patients suffering without being able to help them improve quickly… So, when I saw it, it was so hard.” P08*

#### Similarities with other APPE rotations

The graduates reported that the major similarity between the psychiatric rotation and other rotations was the clinical pharmacists’ role. Like other non-psychiatric rotations, clinical pharmacists are expected to perform medication reconciliation, interview patients, and manage their pharmacotherapy.

*“The similarities are basically what we’re supposed to do… The pharmacist’s role is similar between all of the rotations.” P03*

#### Uniqueness of the APPE in psychiatry

Participants stated they felt the mental health rotation’s setting was unique in different ways. For example, they felt they have witnessed increased interdisciplinary collaboration compared to other rotations, with an opportunity to learn about other mental health providers’ roles.

*“(the) psychiatry rotation was little bit different, more exciting, because they’re applying multidisciplinary teams efficiently. I interviewed many healthcare providers from the team” P02*

Many graduates also noticed that managing patients in the mental health hospital is unique in terms of its stronger focus on factors other than efficacy of the chosen drugs including safety of the medications, social factors such as availability of family or community support and financial status, and patient preferences. One particularly unique aspect was how rounds were carried out. As opposed to bedside rounds observed in non-psychiatric rotations, multidisciplinary (MDT) patient care rounds at the psychiatric hospital are held once or twice per week, whereby patients are invited to the MDT consultation room to discuss their symptoms, treatment, progress, and discharge plans.

*“…the importance of providing a safe, non-judgmental, no-stigma environment and the importance of the social background… not only do we care about the medications but also about the patient holistically… you have to take social factors, preferences, what the patient’s priorities are (into consideration).” P06*

#### Satisfaction

Participants were generally satisfied with their psychiatric-rotation experience. Most participants said that the rotation exceeded their expectations. They reported gaining essential knowledge of mental illnesses and of interacting with patients.

*“It is between meet and above expectation… by the end of that rotation I felt I'm strong enough to be there, interact with those patients, and start managing and giving my interventions… I know how to deal with those cases in terms of skills and knowledge.” P05*

### Theme 3: Graduates’ views on mental health-related stigma

#### Personal perceptions about mental health stigma

Most participants stated they would never stigmatize patients with mental illness.

*“I don't have stigma towards them… I don't judge people based on their condition whether it's psychiatric or not.” P05*

However, some of them reflected on their initial feelings of apprehension and how these perceptions changed to empathy by the end of the rotation.

*“…I was scared for the first week… the (feeling of) being scared became like feeling sorry for them and empathizing with them.” P03*

#### Societal stigma

For the most part, participants perceived the presence of societal stigma against patients with mental illnesses. Patients and their families are often afraid to seek help and take psychiatric medications in fear of judgement.

*“There is a major stigma going on in our society. Even in the psychiatry hospital itself… Some families think that these people have been possessed by Jinn (evil spirits)” P07*

#### Healthcare provider’s stigma

Most participants mentioned that healthcare providers did not stigmatize patients receiving care at the mental health hospital. Nevertheless, stigma still existed. One participant mentioned the story of a provider reluctant to receive his own non-psychiatric prescription from the service’s building, fearing it would be marked on his file that he received medications from the mental health facility.

*“…he didn’t want it on the system (to show) that he got the medication from the mental health facility, so people don’t think he’s crazy.” P01*

### Theme 4: Areas of improvement for the psychiatric rotation experience

#### Logistical barriers

Graduates reported they would spend their rotation in one ward, mostly providing care to adult male patients. Most mentioned the desire to experience managing females or children and adolescents. Some also mentioned they felt they needed more supervision by their preceptors during rounds and interactions with patients.

*“I visited the paediatric psychiatric hospital for one day only and I met only one patient.” P09*

#### Loss of opportunities due to the coronavirus disease 2019 (COVID-19) pandemic

Due to the recent COVID-19 pandemic, students who underwent the psychiatric rotation during 2020 were not able to attend in-person psychiatric-hospital rotations. Instead, rotations were conducted virtually through scheduled online meetings with preceptors and completed at-home assignments. As a result, these students felt their training was incomplete as they were not able to directly see, interact with, or provide care to patients in person.

*“We lost the opportunity of actually seeing psychiatric patients face to face. I would have loved to interview or see how healthcare providers interview [these] patients.” P01*

### Theme 5: Positive impact of the APPE rotation in psychiatry

#### Increased knowledge and awareness of mental health

Some experiences were described by the participants as *“eye-opening”.* For instance, before their mental health rotation, graduates were shocked that electro-convulsive therapy (ECT) is brief, painless, and commonly used in the treatment of refractory patients. Additionally, participants mentioned they were surprised to learn that the presentation and management of each patient could greatly vary even if they have the same diagnosis.

*“Electro-convulsive therapy is something that shocked me. I expected it to be like movies. People will be in pain and … it's just like torturing the patient. However, when I went there, I was surprised. It was very brief.” P08*

#### Influence of the psychiatric rotation on current practice

Graduates reported that the rotation positively influenced their current practice. For the most part, participants perceived themselves to be more confident in providing care to patients with mental illnesses. Some said that it changed their attitudes and made them more empathetic towards patients.

*“I feel more confident on how to actually treat them and manage their medications, their chronic medications. I feel more comfortable talking to them… understanding them.” P08*

## Discussion

To the best of our knowledge, this is the first study to qualitatively investigate the opinions of PharmD graduates on mental health practice after their experience in an APPE psychiatric rotation. Regardless of their familiarity with mental illnesses, the first impressions of the PharmD graduates carried a tone of mixed feelings towards the setting and the patients, which slowly changed as they progressed in the rotation and gained valuable insight of psychiatry practice. Similar feelings of apprehension when starting psychiatric rotations have been reported by students from pharmacy and other healthcare programmes [22–24]. Participants in this study described their PharmD psychiatric-rotation experience as unique, eye opening, and insightful. Observations of the unique presence of interdisciplinary collaboration and holistic pharmaceutical care provision to patients in mental health rotations was also highlighted in other studies and are considered to be an important factor for strengthening pharmacy students’ confidence [12]. Another unexpected experience that emerged from the interviews was about the use of ECT in mental health practice. Before their rotation, many participants believed ECT to be outdated and dangerous, but witnessing it during their training changed their views. Pharmacy students in Ireland had a similar experience after a visit to the mental health hospital, where many realized misconceptions about ECT [25].

This study also contributes to the body of literature in regards to stigma surrounding mental health practice among pharmacy graduates. Overall, studies have shown mixed results, with some finding a strong positive effect of psychiatric rotations in reducing pharmacy students’ stigmatizing attitudes towards the provision of care to people with mental illnesses, while others reported an opposite or no significant results [11, 12, 26–28]. When discussing their views on mental health stigma, participants in our study recalled their feelings of apprehension, and even fear, before starting the APPE rotation in psychiatry. These feelings are understandable considering the large body of evidence in regards to stigmatizing attitudes towards people with mental illnesses being largely embedded in the Arab culture [7]. In a previous study at different colleges in QU, a significant societal stigma was reported by the university students, with over 50% believing that *“mental illness is a punishment from God”* [29]. Although this stigma was not apparent in relation to the provision of care, participants in our study still expressed potentially stigmatizing personal biases in their interactions with patients, such as associating mental illness with danger and crimes, minimizing patient interaction, or using stigmatizing language. Similar findings in relation to social stigma has also been reported by pharmacy students in Canada [30]. Nevertheless, the fact that PharmD graduates in our study recognized and rejected these stigma sources after completing the rotation, including their own internal and implicit biases, is a promising finding and aligns well with the results of other studies which suggest that completion of an inpatient psychiatric APPE decreased stigma among pharmacy students [12]. Focused stigma-related discussions in rotations may further prepare future pharmacists for pharmaceutical care provision to people with mental illnesses. An essential focus of these discussions should include orienting students to using appropriate language to describe mental illness and those who are affected. During the interviews participants often used disease-first language such as “schizophrenic” or “mentally ill”, described patients as “suffering” from a mental illness, or used derogatory terms such as “crazy”. Studies have shown that using scientifically accurate language and terms to describe these conditions makes a significant difference for the people experiencing them, and can help in reducing mental health related [31, 32].

One of the barriers observed by participants to have affected their confidence when providing care to patients with mental illnesses was their limited knowledge and familiarity with mental illnesses. For many PharmD graduates, the BSc programme itself provided some basic knowledge. However, many felt it was insufficient, leaving them unprepared for practicing in a mental health facility or managing the diversity of mental illnesses presentations —both skill- and emotional-wise. Similarly, a study amongst Nigerian pharmacy students noted the majority of PharmD and BSc students felt the undergraduate curriculum did not adequately prepare them to manage mental health patients and reported that there was insufficient exposure to real practical experiences [13]. There are also reports in the literature on how these educational gaps have been addressed by some pharmacy schools, such as: including classroom instruction that foster better understanding of mental illnesses through in-depth discussions, sessions instructed by mental healthcare professionals, counselling sessions with simulated patients, and including interactions with mental health consumers [9, 33, 34]. Other barriers emerged due to some of the participants’ APPE experience during the COVID-19 pandemic, which required a shift to virtual rotations. Despite the rotation organizers’ efforts, some participants still felt essential elements of the experience were missing. As such, the integration of telemedicine should be considered into the contingency plans for psychiatric rotations in case of future emergencies [35, 36].

### Limitations and strengths

Unlike existing literature investigating pharmacy students’ attitudes after mental health rotations, this study offers a qualitative exploration of PharmD graduates’ opinions on their experiences after a psychiatric APPE clinical rotation. It does not only provide insight of their opinions on the rotation, but also investigates the rotation’s impact on their pharmaceutical care provision to individuals with mental illnesses. As the CPH-QU APPE rotation in psychiatry is relatively new, a limited sample of participants was available for recruitment. In addition, some graduates experienced the rotation years ago, creating potential for recall bias or diversity. This was minimized using a detailed semi-structured interview guide with open-ended questions and probing statements. Lastly, some of the research members have experience with the rotation or were involved in its design. Therefore, only individuals with no such experiences were involved in the initial data collection and analysis—reducing the possible researcher bias. Similarly, as only undergraduate research students conducted the interviews, asymmetrical power dynamics were minimized and acquiescence bias was reduced [37].

### Future research

The results of this study provide the basis for future research in several directions. Future studies could explore perceptions of PharmD graduates in other countries. It might also be interesting to compare attitudes towards mental health practice against those who did not complete a psychiatric APPE rotation. Additionally, it might be worthwhile to implement and evaluate creative interventions to enhance mental health awareness in undergraduate BSc pharmacy programmes to make the transition to post-BSc PharmD APPE psychiatric rotations less intimidating.

## Conclusion

This study investigated PharmD graduates’ experiences during a psychiatric clinical rotation and explored their opinions towards mental health, patients, and practice. Findings from this study support that mental health rotations are crucial in the preparation of PharmD graduates for pharmaceutical care provision to people with mental illnesses. It is essential that educators enhance current undergraduate and PharmD curricula to ease transition into practice through reducing stigma, increasing knowledge, and providing familiarity with the diversity of mental health presentations.

### Supplementary Information

Below is the link to the electronic supplementary material.

Supplementary file1 (DOCX 21 KB)
