# Supplementary material for: Opinions and experiences on the provision of care to people with mental illnesses: a qualitative study with Doctor of Pharmacy graduates after a rotation in psychiatry
Source: Int J Clin Pharm. 2023 Oct 5;45(5):1223–30. doi: 10.1007/s11096-023-01646-1 (PMC10600030; doi:10.1007/s11096-023-01646-1)
Supplement: Supplementary file 1 — Supplementary file1 (DOCX 21 KB) [file 11096_2023_1646_MOESM1_ESM.docx]

**Supplementary Material**

**Semi-structured Interview Guide**

1. Prior to your rotation at the psychiatric hospital, did you have contact with people or patients who had mental health disorders? Tell me a bit about this experience?

2. What were your first impressions when you started the rotation at the psychiatric hospital? Tell me a bit on what went well and what did not.

3. Did you feel you were adequately prepared for what you encountered during your mental health rotation?

4. Did anything in your mental health rotation particularly surprise you? Tell me a bit more about that experience.

5. What aspects of your mental health rotation were different/similar from the other rotations you have completed during your PharmD program?

6. Overall, did the mental health rotation meet your expectations? Tell me a bit more about how it did/did not exceed your expectations.

7. If you were to repeat this experience, what would you do/not do to make the rotation better?

8. In retrospect, what was the most valuable thing that you took away from this experience?

9. How did your participation in the mental health rotation influence your current practice. Did this rotation influence your approach to patient care in any way? Please describe.

10. Did this rotation affect you personally in any way?

11. Any other comments you would like to bring forward regarding your PharmD mental health rotation experience?

Thank you so much for being here and for taking this interview with me.
